# Clinical and Neuroimaging Characteristics of Mild Cognitive Impairment Patients with Positive Central Nervous System Autoantibodies

**DOI:** 10.1002/alz70857_097138

**Published:** 2025-12-24

**Authors:** Heya Luan, Jin Gong, Wenxian Sun, Pin Wang, Xiaodong Han, Chang Xu, Cuibai Wei

**Affiliations:** ^1^ Xuanwu Hospital, Capital Medical University, Beijing, Beijing, China; ^2^ College of Integrated Traditional Chinese and Western Medicine, Changchun University of Chinese Medicine, Changchun, Jilin, China; ^3^ Innovation Center for Neurological Disorders and Department of Neurology, Xuanwu Hospital, Capital Medical University, National Clinical Research Center for Geriatric Diseases, Beijing, Beijing, China

## Abstract

**Background:**

Central nervous system autoantibodies have been identified in mild cognitive impairment (MCI) and dementia. This study firstly provides a comprehensive description of the clinical and neuroimaging characteristics of MCI with autoantibodies.

**Method:**

In this retrospective study, MCI patients were continuously recruited from a memory clinic and tested for central nervous system autoantibodies using cell‐based assays (CBA). Clinical characteristics were compared between antibody‐positive and antibody‐negative groups. Furthermore, patients underwent magnetic resonance imaging (MRI) to evaluate cortical atrophy, ^18^F‐Fluorodeoxyglucose positron emission tomography (FDG‐PET) to analyze glucose metabolism patterns and Pittsburgh Compound B positron emission tomography (PIB‐PET) to assess amyloid deposition patterns. The standardized uptake value ratio (SUVR) was calculated in the whole brain and 90 regions of interest (ROIs) defined by the Automated Anatomical Labeling (AAL) atlas. The relationship between the presence of autoantibodies and SUVR was also analyzed.

**Result:**

Ninety‐seven MCI patients were included, with 10.3% positive for autoantibodies, predominantly intracellular antibodies. Only abnormal tumor markers are more frequent in the antibody‐positive group. The antibody‐positive group exhibited higher whole‐brain glucose metabolism in FDG‐PET, predominantly in the frontal lobe, while no significant difference was observed in MRI or PIB‐PET. Furthermore, a positive correlation was also found between glucose metabolism and presence of autoantibodies.

**Conclusion:**

Abnormal tumor markers and increased frontal lobe metabolism on FDG‐PET may serve as potential indicators for identifying MCI with autoantibodies. The underlying pathogenesis could involve a pathogenic mechanism distinct from amyloid deposition.

